# The Role of Nutrients in Maintaining Hematopoietic Stem Cells and Healthy Hematopoiesis for Life

**DOI:** 10.3390/ijms23031574

**Published:** 2022-01-29

**Authors:** Yuko Tadokoro, Atsushi Hirao

**Affiliations:** 1 Division of Molecular Genetics, Cancer Research Institute, Kanazawa University, Kakuma-machi, Kanazawa 920-1192, Japan; tadokoro@staff.kanazawa-u.ac.jp; 2 WPI Nano Life Science Institute (WPI-Nano LSI), Kanazawa University, Kakuma-machi, Kanazawa 920-1192, Japan

**Keywords:** hematopoietic stem cell, nutrient, catabolism, anabolism, dietary intervention

## Abstract

Nutrients are converted by the body to smaller molecules, which are utilized for both anabolic and catabolic metabolic reactions. Cooperative regulation of these processes is critical for life-sustaining activities. In this review, we focus on how the regulation of nutrient-driven metabolism maintains healthy hematopoietic stem cells (HSCs). For this purpose, we have examined the metabolic regulation of HSCs from two perspectives: (1) the control of intracellular metabolism by the balance of anabolic and catabolic reactions; and (2) the control of organismal metabolic status and hematopoiesis by dietary intake of nutrients. Critical roles of catabolic regulators in stem cell homeostasis are conserved in several types of tissues, including hematopoiesis. These catabolic signals are also major regulators of organismal lifespan in multiple species. In parallel, changes to nutrients via alterations to dietary intake affect not only an organism’s metabolic state but also the behavior of its stem cells. While the molecular mechanisms involved in these two aspects of nutrient function may not necessarily overlap, a deeper understanding of these phenomena will point to new avenues of medical research and may furnish new agents for improving human health care.

## 1. Introduction

Nutrients are substances in food that are essential for biological activity in organisms and include carbohydrates, lipids, proteins, vitamins, and minerals. Nutrients are converted by the body into smaller molecules that are utilized for the set of life-sustaining chemical reactions called metabolism. Collectively, reactions that break down food or fuel to obtain energy are called catabolism, whereas the reactions by which larger molecules are synthesized from smaller ones are called anabolism. Anabolic reactions consume the energy produced by catabolic reactions, such that cooperative regulation of both processes is critical for maintaining life.

**Figure 1:**
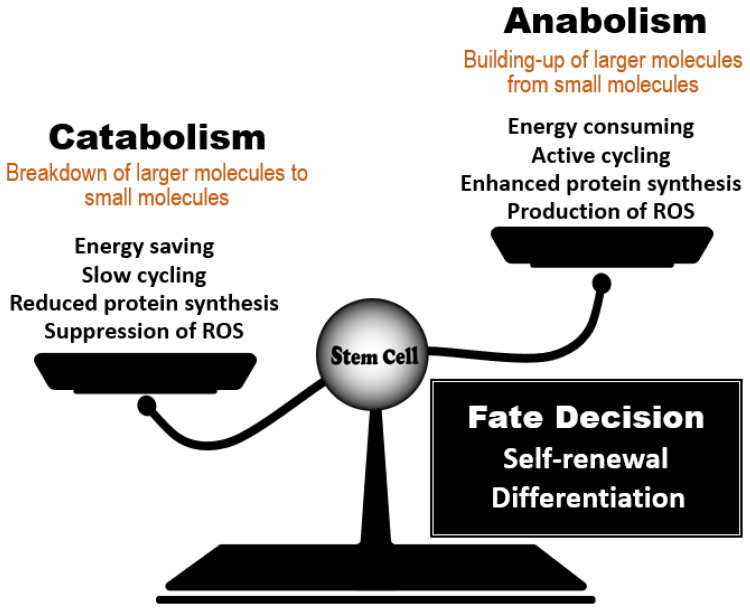
Regulation of stem cell fate decisions by the anabolic/catabolic balance. Stem cell behavior is influenced by a balance between regulators of catabolic and anabolic metabolic reactions. Dominance by catabolic regulators, which act to reduce energy use, protein synthesis and ROS accumulation, favors stem cell quiescence and the steady-state. When anabolic regulators gain dominance, active cycling by a stem cell increases its energy consumption and protein synthesis, enabling the cells to undertake the decision to either self-renew or differentiate. The anabolic/catabolic balance thus has a major influence on stem cell homeostasis.

Hematopoietic stem cells (HSCs) have unique metabolic characteristics that contribute to the maintenance of their homeostasis in blood [1]. In an animal at steady-state, the majority of HSCs within the bone marrow (BM) niche maintain low levels of reactive oxygen species (ROS), ATP production, and protein synthesis (translation); these properties are associated with a state of cell cycle arrest called quiescence or dormancy. Debate has raged for a long time over whether metabolic status controls HSC fate, i.e., whether this relationship is causative or not. It is now evident that nutrient-sensing molecules that govern the balance between anabolic and catabolic processes play critical roles in HSC maintenance by controlling their intracellular metabolism (Figure 1). Knowledge gained over the past 15 years has generated three fundamental conclusions in this field. First, HSCs depend on catabolic regulators to remain healthy. Accumulating evidence has demonstrated that the acquisition of catabolic status (as supported by autophagic and lysosomal activity), coupled with reduced anabolic signaling, maintains HSC function and prevents the development of phenotypes of aging. Second, the essential roles of catabolic regulators in HSC homeostasis are conserved in other types of tissue stem cells that remain dormant in animals at steady-state [2]. Third, these catabolic signals are major regulators of organismal lifespan in multiple species [3].

Another important issue in the relationship between nutrients and healthy HSCs is an organism’s dietary intake. Dietary interventions such as dietary restriction (DR), or the feeding of a ketogenic or high-fat diet (HFD), markedly affect an organism’s metabolic state and the behavior of its stem cells [4]. There is evidence showing that DR (such as prolonged fasting) contributes positively to HSC functions, whereas the prolonged consumption of a HFD induces aberrant HSC self-renewal. Compared to our knowledge of the effects of nutrients on other stem cell types such as intestinal stem cells (ISCs), our understanding of the influence of nutrients on HSCs is much more limited. However, it is clear that signaling molecules involved in dietary interventions have a significant impact on both HSCs and the cells that surround them in the BM niche.

In this review, we focus on the above two aspects of nutrient-driven metabolic regulation required to maintain healthy HSCs. In the first section, we discuss the control of intracellular metabolism by the balance between anabolism and catabolism and how it helps to maintain quiescent HSCs. In the second section, we present an overview of recent findings on how changes to organismal metabolic status caused by altered dietary intake of nutrients affect HSC homeostasis. While the molecular mechanisms involved in these two aspects of nutrient function may not necessarily overlap, a better understanding of both sets of phenomena may point to ways to improve human health by ensuring healthy HSC homeostasis for life.

## 2. Effect of the Anabolic/Catabolic Balance on Stem Cell Homeostasis

### 2.1. Commonality of Nutrient-Sensing Regulators

Several types of tissue stem cells, including HSCs, experience long periods of quiescence or dormancy throughout the life of the host organism [2]. In HSCs, quiescence blocks the cell cycle and maintains the cells in an undifferentiated state that protects them against various insults, including DNA breaks and oxidative stress [1]. Similarly, resident satellite cells in muscle, known as muscle stem cells (MuSCs), are quiescent in an organism at steady-state [5]. In response to injury, MuSCs enter the cell cycle to regenerate the skeletal muscle tissue and replenish the MuSC pool. The same applies to adult neural stem cells (NSCs) in the subventricular zone (SVZ) of the brain, which are preserved in a dormant state in healthy animals [6]. Most adult NSCs cells are derived from a quiescent subpopulation of embryonic NSCs and differentiate into interneurons that migrate into the olfactory bulb.

The catabolic regulators required to maintain the dormancy of tissue stem cells are shared in animals. The regulators can also prolong lifespan or delay aging in multiple species, including yeast, worms, flies, and mice [3]. Conversely, stem cells in the intestinal epithelium and epidermis are actively cycling, and this cycling is vital to their homeostasis [2]. These differences indicate that the regulation of stem cell biology is not simple. In the sub-sections that follow, we review several evolutionarily conserved signaling pathways that are linked to catabolic status and modulate organismal longevity, but which also are important common regulators of the functions of HSCs, NSCs and MuSCs.

### 2.2. Catabolic Regulators That Maintain Stem Cell Quiescence

#### 2.2.1. Autophagy Regulators

Autophagy is a conserved catabolic system that degrades cellular components which have become engulfed in autophagosomes [7]. Autophagy contributes to maintenance of routine cellular functions, but it is induced to a high level in response to insults such as oxidative stress, infection, and starvation. Autophagy is thus a survival mechanism that maintains cellular homeostasis by eliminating unwanted components such as damaged organelles, protein aggregates, and intracellular bacteria. Autophagy also supplies an energy source under stress conditions.

Autophagy occurs in a series of steps that are controlled by various autophagy-related genes (*Atgs*). These steps include initiation, formation of a phagophore structure, maturation of an autophagosome, fusion of the autophagosome with a lysosome, degradation of the engulfed entity, and recycling of nutrients. The *Atg5* and *Atg7* genes are involved in elongation of the autophagosome membrane. When the Vav-Cre-loxP system was used to delete these genes specifically in murine hematopoietic cells during the fetal period, the animals suffered from weight loss, severe anemia, and a reduction in HSCs that resulted in death [8,9,10,11,12]. However, mice in which the gene was deleted at either the juvenile or adult stage appeared largely healthy. Detailed analysis of HSC functions after birth showed that, although Atg5 deficiency induced no obvious abnormalities at P0, significant defects arose at P7 [12]. In addition, induction of *Atg5* deletion at P5 did not result in remarkable abnormality in hematopoiesis. These data suggest a critical role for autophagy in protecting HSCs against insults during the early neonatal stage, which is essential for healthy long-term hematopoiesis. In adult, HSCs with loss of *Atg12*, which is also involved in elongation of the autophagosome membrane, show impaired functions, increased mitochondrial content, and a bias toward myeloid differentiation [13,14]. At the signaling level, autophagy is induced rapidly upon starvation via a gene expression program that is driven by forkhead transcription factor FoxO3 (FoxO3). Thus, autophagy contributes to the HSC maintenance of their low metabolic state and quiescence by degrading and clearing active healthy mitochondria at steady-state.

**Figure 2:**
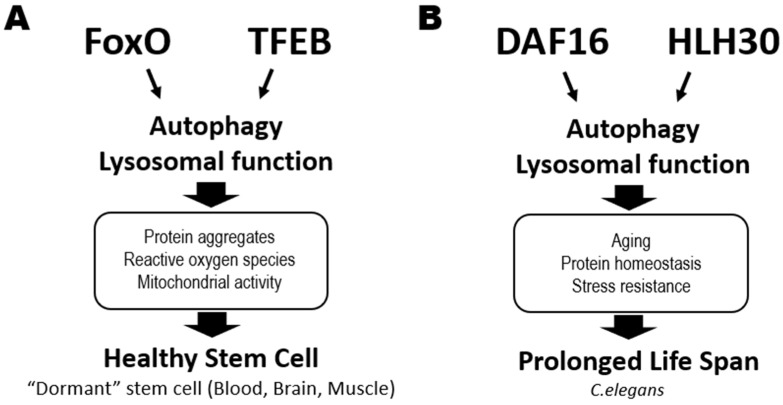
Critical roles of catabolic regulators in organismal life extension and the maintenance of healthy stem cells. (**A**) In mammalian stem cells, activation of the FoxO and TFEB transcription factors triggers autophagy and lysosomal functions that help to maintain tissue stem cell quiescence or dormancy in an animal at steady-state. This process reduces the accumulation of protein aggregates and ROS and suppresses mitochondrial activity in stem cells in blood (HSCs), brains (NSCs) and muscle (MuSCs), sustaining their good health. (**B**) DAF16 and HLH30 are the homologs of FoxO and TFEB, respectively, in *C. elegans*. These transcription factors also cooperate to regulate genes involved in autophagy and lysosomal functions, which prolong a worm’s lifespan by maintaining protein homeostasis and increasing stress resistance.

Autophagy is also involved in the “aging phenotypes” exhibited by HSCs isolated from aged animals [13,14]. Mice unable to carry out autophagy as adults develop hematopoietic phenotypes resembling those of aged normal mice, such as increased cellularity and a skewed ratio of myeloid versus lymphoid cells in peripheral blood. Thus, regulation of metabolic status via autophagy is crucial for HSC homeostasis throughout the entire life of an animal (Figure 2A).

In NSCs, autophagy plays a critical role in maintaining quiescence by reducing protein aggregates in a FoxO3-mediated manner and by decreasing ROS in mitochondria [15]. During aging, NSCs show a reduction in lysosome content and an increase in protein aggregates, suggesting that autophagy-mediated lysosomal activity is critical for the control of the latter [16]. Among MuSCs, autophagic flux is significantly elevated in cells from younger mice but reduced in MuSCs from aged animals [17]. Loss of Atg7 impairs mitophagy and elevates ROS, leading to a loss of the MuSC pool. Pertinently, restoring autophagy improves the function of MuSCs from aged mice.

Collectively, these data indicate that autophagy makes a major contribution to stem cell quiescence, maintenance, and prevention of premature aging.

#### 2.2.2. Transcription Factor EB (TFEB)

Gene transcription associated with catabolic processes is induced by the nuclear translocation and consequent activation of the MiT/TFE family members, such as transcription factor EB (TFEB), a master modulator of autophagy and lysosomal biogenesis (Figure 2A, right). In *C. elegans*, activation of HLH30, its TFEB homolog, prolongs worm lifespan by promoting autophagy and lysosome functions [18] (Figure 2B). Accordingly, TFEB agonists also extend *C. elegans* lifespan [19]. In humans, lysosome functions are regulated dichotomously by TFEB and MYC to balance catabolic and anabolic processes required for activating HSCs and guiding their lineage fate [20]. The TFEB-mediated endolysosomal pathway promotes HSC quiescence and self-renewal, reinforcing the theory that catabolic processes control HSC fate determination. Similarly, TFEB deficiency or chemical inhibition of lysosomal degradation impairs NSC quiescence and function [21]. Quiescent NSCs exhibit large lysosomes containing insoluble protein aggregates, have higher lysosomal activity, and degrade activated EGF receptors by endolysosomal degradation [16,22]. Although the role of lysosomal activity in MuSC homeostasis has yet to be elucidated, the commonalities among HSCs and NSCs suggest that it would not be surprising to find a crucial role for TFEB in this stem cell type as well.

#### 2.2.3. FoxO Family

As mentioned above, FoxO family members are vital for catabolic regulation in tissue stem cells. In *C. elegans*, reduced insulin/IGF-1 signaling extends lifespan in a process mediated by DAF16 (FoxO homolog) [23]. DAF16 and HLH30 (TFEB homolog) cooperatively regulate genes related to aging, protein homeostasis, and stress resistance, helping to prolong lifespan in a harsh environment [24] (Figure 2B). In mammals, FoxO1, FoxO3, FoxO4 and FoxO6 are all downstream targets of PI3K-AKT signaling [25]. In the absence of cellular stimulation by growth factors or insulin, FoxOs localize in the nucleus and activate their transcriptional targets. When a growth factor or insulin binds to the appropriate cell surface receptor, AKT is activated and directly phosphorylates FoxOs, resulting in their nuclear exclusion and degradation in the cytoplasm. Metabolic or oxidative stress can also induce nuclear localization of FoxOs and their transcriptional activity. In HSCs, it is FoxO1 and FoxO3 that are mainly expressed and localized in the nucleus under conditions of AKT inactivation [26,27]. Deficiency of FoxO3 or FoxO1/3/4 in HSCs results in defective quiescence and decreased long-term repopulating capacity [28,29,30]. This impaired HSC quiescence is linked to elevated ROS and modified expression of genes involved in either cell cycle arrest, such as *Cdkn1b*, *Ccng2*, *Cdkn1c* and *Cdkn1a*, or redox regulation, such as *SOD* [28,29,30]. However, the roles of FoxOs in ROS-dependent regulation of HSC functions is complex. One study reported that abnormalities of FoxO1/3/4-deficient HSCs could be rescued by the anti-oxidative reagent N-acetyl cysteine (NAC) [29], whereas another group found that NAC did not restore the functions of FoxO3-deficient HSCs [31]. In HSCs from obese mice, FoxO proteins become insensitive to their normal upstream regulators such as AKT, suggesting that hyperglycemia can directly alter the AKT-FoxO axis in these cells [32]. Thus, FoxOs are important for the maintenance of healthy HSCs in animals both at steady-state and in those under environmental stress.

FoxOs are also critical regulators in pluripotent stem cells. In embryonic stem (ES) cells, FoxO1 activates the expression of the pluripotency genes *Oct4* and *Sox2*, promoting ES cell self-renewal capacity [33]. FoxO1 also controls ES pluripotency by directly regulating the expression of core autophagic machinery genes [34]. FoxO3 protects ES cells from hyperglycemic stress by inducing upregulation of SOD2, catalase, p21 and p27, thereby promoting ROS detoxification and cell cycle arrest [35]. In NSCs, loss of FoxO1/3/4 or FoxO3 leads to defects in quiescence and undifferentiated status. Mechanistically, FoxOs control genes regulating the cell cycling and redox state of NSCs. In MuSCs, FoxOs are again critical for quiescence and prevention of premature aging. Niche-derived IGF1-dependent AKT activation negatively controls MuSC functions mediated by FoxOs [36]. While nuclear localization of FoxO3 is observed in Pax7^+^ MuSCs, loss of FoxO3 impairs quiescence, leading to MuSC exhaustion [37]. An intriguing study has revealed how FoxOs act to prevent of premature aging of MuSCs. There are two types of quiescent MuSCs: those of a “genuine state”, which exhibit stemness, and those of a “primed state”, which commit to myogenic differentiation [36]. Extremely old mice lose “genuine” MuSCs and gain “primed” MuSCs. FoxO1/3/4 deficiency causes a similar loss of “genuine” MuSCs, causing regenerative failure. These data indicate that stem cell aging is controlled by FoxOs through their regulation of catabolism and stress responses.

Collectively, these observations link stem cell maintenance and function to the coordinated cooperation of numerous factors governing intracellular nutrients, including catabolic pathways, autophagy, and TFEB and/or FoxOs activation.

#### 2.2.4. Anabolic Regulators That Control HSC Self-Renewal

One characteristic that is unique to quiescent HSCs is downregulation of protein synthesis [38,39]. This observation suggests that suppression of anabolism may be a critical hallmark of healthy HSCs. In general, protein synthesis in cells is strictly controlled by mTOR complex 1 (mTORC1), which is one of two functionally different protein complexes containing the vital kinase mTOR [40]. mTORC1 has three core components: mTOR, “mammalian lethal with SEC13 protein 8” (mLST8, also known as GβL), and the scaffold protein “regulatory-associated protein of mTOR” (RAPTOR). mTORC1 phosphorylates numerous protein substrates, including S6K1 and eIF4EBPs, to regulate protein translation. mTORC1 also upregulates the selective protein translation of mRNAs with 5’TOP structure. Once activated by mTORC1-mediated phosphorylation, S6K1 phosphorylates ribosomal protein S6, a component of the 40S ribosomal subunit, to upregulate rRNA transcription and promote the translation elongation of spliced transcripts. Quiescent HSCs show profound inhibition of S6 and 4EBP phosphorylation [41], confirming the downregulation of protein synthesis in these cells [42].

The regulation of mTORC1 is critical for HSC functions. Induction of mTORC1 hyper-activation by Tsc1 deletion or Rheb overexpression causes HSC depletion that is associated with cell cycle progression and mitochondrial activation [43,44,45]. The suppression of mTORC1 signaling in quiescent HSCs depends on Pten, which is a negative regulator of the PI3K-AKT pathway. Deletion of Pten in mice induces hyperactivation of mTORC1 in HSCs, aberrant cell cycle progression, and upregulation of p16Ink4A and p53, resulting in HSC depletion [46]. These abnormalities of HSCs can be reversed by the mTOR inhibitor rapamycin, confirming that mTORC1 hyperactivation leads to HSC failure in vivo. Raptor deficiency shows mTORC1 inactivation, and these cells gradually lose their capacity for hematopoietic reconstitution [47]. Therefore, mTORC1 function appears to be crucial for HSC maintenance regardless of Pten deficiency.

While hyperactivation of mTORC1 induces severe defects in HSC function, lower levels of mTORC1 activity are necessary for HSC self-renewal. Similar to Raptor, Rheb1 is an activator of mTORC1. Loss of Rheb1 suppresses mTORC1 activity, but the level of mTORC1 activity that remains in Rheb1-deficient cells is significantly greater than that in Raptor-deficient cells [48]. While the total number of HSCs as defined by marker phenotype is increased by deficiency of either Raptor or Rheb1, competitive reconstitution assays in vivo have demonstrated a clear difference in the functionality of Raptor-deficient vs. Rheb1-deficient HSCs [48]. Loss of Raptor, but not Rheb1, reduces HSC competitiveness in vivo, suggesting that HSCs that expand due to Raptor deficiency lose their stemness, whereas Rheb1 deficiency induces the expansion of HSCs that retain their stemness. These data suggest that the fine-tuning of moderate levels of mTORC1 activity is needed to preserve HSC self-renewal activity in vivo. Consistent with this finding, rapamycin-mediated inhibition of mTORC1 has made it possible to perform ex vivo HSC expansion for both mice and humans [49,50]. Combining rapamycin with a GSK-3 inhibitor further increases the number of functional HSCs, indicating a role for nutrient-sensing in the maintenance of HSCs in culture [51]. Rapamycin treatment also reverses the natural aging-related decline in HSC function in normal mice [52]. Thus, strict control of mTORC1 activity is the key to maintaining or expanding healthy HSC populations in vitro and in vivo.

Taken together, the findings presented in the first part of this review establish the importance of both catabolic and anabolic regulatory elements in HSC biology.

## 3. The Effects of Dietary Interventions on Stem Cell Homeostasis

### 3.1. Beneficial Effects of Dietary Restriction and Prolonged Fasting on HSC Functions

Dietary restrictions (DRs) have been shown to prolong lifespan by delaying aging in multiple species [53,54]. This observation has led to the hypothesis that such dietary interventions can also prevent the aging of HSCs and thus promote their preservation in a healthy state. In general, DR means reduced intake of either total dietary calories or one or more specific components of the diet. Intermittent and periodic fasting are also forms of DR. In the laboratory, differences in experimental DR protocols can generate different results. For example, Lazare et al. investigated the effects of caloric restriction (CR) on HSC function by feeding C57BL/6 (B6) mice on a diet containing 30% fewer calories than a control diet. These researchers showed that lifelong CR in mice did not have any impact on aging-associated HSC phenotypes [55]. However, Tang et al. reported that a 30% DR regimen, in which mice were fed only 70% of the amount of food consumed by ad libitum-fed mice, prevented HSCs from displaying aging phenotypes. The DR-treated mice in this study possessed fewer of the CD150^high^ myeloid-biased HSCs that increased in number with age compared to mice on the control diet. In this case, DR also conferred on HSCs increased dormancy and enhanced reconstitution ability compared with HSCs isolated from ad libitum-fed mice [56]. In addition to effects on HSCs, this form of DR suppressed lymphopoiesis while enhancing erythropoiesis and myelopoiesis in early progenitor cells. Mechanistically, DR suppressed expression levels of IGF-1, IL-6 and IL-7, which could be rescued by feeding the ad libitum diet to DR-treated mice. All three of these molecules proved to be involved in one or more DR-induced hematopoietic changes. For example, enhancement of quiescence of HSCs by DR-treatment was normalized by administration of IGF-1, but impaired lymphopoiesis was not rescued. In contrast, injection of IL-6 or IL-7 was able to rescue lymphopoiesis in DR-treated animals. These results indicate that DR affects hematopoiesis in a cell type-specific manner, and that multiple factors are involved in exerting DR-related effects.

When normal mice are treated with prolonged cycles of fasting and feeding, beneficial effects are conferred on HSCs. For example, Cheng et al. subjected mice to repeated cycles of fasting for 48 h followed by feeding for 12–14 days and examined the effects on hematopoiesis [57]. This group reported that prolonged fasting led to HSC expansion, protection of HSCs against chemotoxicity, and promotion of HSC regeneration capacity coupled to normal differentiation ability. Thus, limitation of nutrient intake by DR or prolonged fasting has a positive effect on HSC functions.

**Figure 3:**
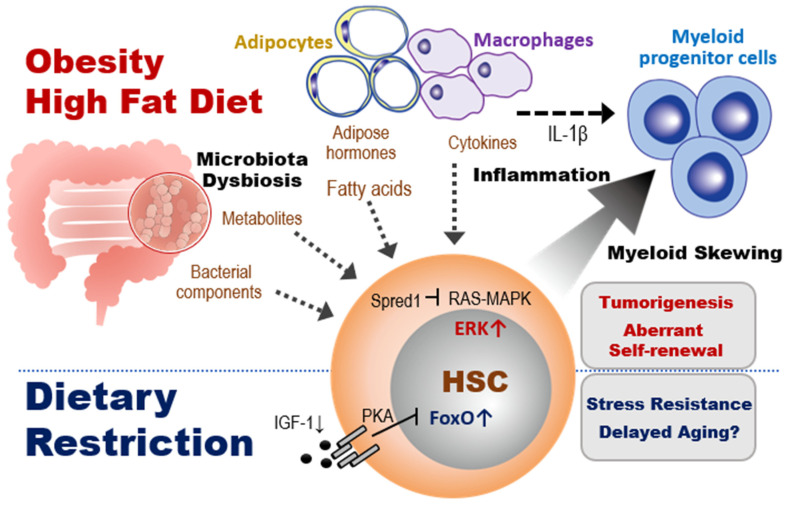
Impacts of dietary interventions on HSCs and myeloid progenitors. Top section: Obesity or HFD alters the spectrum of bacterial and adipocyte products and macrophage-produced cytokines that act on HSCs in the BM. In the presence of inflammation, these changes promote macrophage secretion of cytokines that cause HSCs to undergo abnormal self-renewal and a skewing to myeloid progenitor differentiation. In tumor-prone mice such as Spred1-deficient animals, in which RAS-MAPK-mediated tumor suppression is lost, HFD triggers aberrant myelopoiesis leading to the development of myeloproliferative neoplasm-like diseases. Bottom section. In contrast to obesity, a DR such as fasting reduces IGF-1 and PKA signaling, which activates FoxO1. This FoxO1 activation promotes the quiescence and stress resistance of HSCs and thereby prolongs host lifespan.

IGF-1 is well known as an aging-associated molecule in several tissues [54], but its physiological role in HSC behavior appears complicated. Cheng et al. found that the beneficial effects of prolonged fasting cycles on HSC function were mediated by a reduction in IGF-1/PKA signaling, which induced upregulation of FoxO1 expression and downregulation of G9a [57] (Figure 3, bottom). A recent report has shown that BM levels of IGF-1, which is expressed mainly by mesenchymal cells, decreased with age [58]. This reduction of IGF-1 in the BM microenvironment then induced myeloid-biased hematopoiesis that could be reversed by IGF-1-mediated stimulation of HSCs. Thus, these data appear to conflict with those from the study mentioned above. It is possible that feeding after fasting may induce sudden and significant changes to IGF-1 levels that alter the metabolic dynamics affecting HSC behavior. Fine-tuning of the IGF-1 signaling pathway within an optimum range may be important for maintaining healthy HSCs. A more detailed dissection of the effects of IGF-1 signaling on HSC capacities for self-renewal and balanced differentiation are needed.

### 3.2. The Effects of Obesity on HSC Homeostasis

Obesity caused by a sedentary lifestyle and unhealthy eating habits leads to inflammatory myelopoiesis in mice and humans. Because chronic, low-grade inflammation due to obesity exacerbates age-related diseases, it has been assumed that obesity may also accelerate aging-related defects in stem cells. However, the effects of obesity on HSCs remain unclear.

Previous studies using several different models of obesity, including leptin-deficient *ob* mice, leptin receptor-deficient *db* mice, and mice fed a HFD, have indicated that obesity increases numbers of myeloid-committed progenitor cells, including multipotent progenitors (MPPs), common myeloid progenitors (CMPs), and granulocyte and macrophage progenitors (GMPs) [59,60,61,62,63]. In addition, obesity induces an aberrant BM microenvironment [64,65]. The increase in myeloid-committed progenitor cells in obese mice is associated with adipocyte accumulation in the BM, and it is now clear that BM adipocytes can regulate HSC functions. For example, after BM injury by 5-fluorouracil (5-FU) or cyclophosphamide treatment, adiponectin deficiency inhibited the activation of HSCs by attenuating mTORC1 signaling pathway and delayed hematopoietic recovery [66]. The data suggested that adiponectin promotes hematopoietic regeneration by accelerating the entry of HSCs into the cell cycle. Furthermore, BM adipocytes are an important source of stem cell factor (SCF) after irradiation or 5-FU treatment, and BM adipocyte-derived SCF promotes the maintenance of HSCs and hematopoietic regeneration [67]. These observations show a positive role for at least some effects of adipocytes on HSC function.

In contrast to the above, other studies have suggested that the accumulation of adipocytes within the BM during obesity negatively regulates HSC functions. For example, Ambrosi et al. showed in mice that aging and obesity reprogram the mesenchymal lineage to give rise to adipogenic lineage cells, resulting in expansion and accumulation of BM adipocytes. They also found that BM adipocytes reduced HSC reconstitution ability in a transplant model with isolated BM adipocytes [68]. Others have demonstrated that, in addition to BM adipocytes, other adipose tissue cells are involved in promoting myeloid-biased hematopoiesis during obesity. Nagareddy et al. reported that monocytosis in obesity was associated with infiltration of macrophages into adipose tissue [60]. Transplantation assays of adipose tissue revealed that obesity due to *ob* mutation or HFD promoted NLRP3 inflammasome-dependent IL-1β production by adipose tissue macrophages via the TLR4-MyD88 signaling pathway (Figure 3, top). IL-1β derived from adipose tissue macrophages stimulates the proliferation of CMPs and GMPs, resulting in enhanced myelopoiesis. These observations show a negative role for at least some effects of obesity-induced adipocyte expansion on hematopoiesis.

While obesity appears to affect mainly myeloid-committed progenitor cells, the capacity of HSCs for total long-term reconstitution is also influenced by obesity-related factors [59]. Whereas a primary transplantation of HSCs from *db*/*db* or HFD-induced obese mice resulted in significantly increased chimerism in the recipients, a secondary transplantation of donor-derived HSCs revealed a great reduction in their reconstitution ability. This obesity-induced defect in HSCs depended on upregulated expression of the transcriptional repressor *Gfi1*, which was induced by the increased ROS levels in HSCs. *Gfi1* expression in HSCs can also be induced by aging or acute/chronic inflammation but these effects are reversible. In contrast, the obesity-induced defects in HSCs were irreversible. This obesity-specific regulation of HSC functions remains under investigation.

### 3.3. The Effects of HFD on HSC Homeostasis

In general, a HFD includes animal fats that are rich in undesirable saturated fatty acids. Consumption of a HFD reduces an organism’s insulin sensitivity and the efficiency with which glucose enters the tissues. These effects of HFD alter organismal metabolic status by modulating multiple factors, affecting hematopoiesis (Figure 3, top left).

Hermetet et al. reported that a short term consumption of HFD induces exhaustion of HSCs mediated by modulation of lipid raft organization [69]. Lipid rafts are cholesterol-enriched patches located in the plasma membrane, which play a critical role in dormancy of HSCs mediated by TGF-β signaling [27,70]. While lipid rafts were distributed across the surface of HSC isolated from mice fed a normal diet (ND), they were clustered on HSC from mice fed a HFD, associated with down-regulation of TGF-β signaling. Since TGF-β injection restored impaired HSC functions induced by a HFD, they concluded that consuming a HFD induces alteration of the TGF-β signaling-mediated HSC quiescence, leading to exhaustion of HSCs. Although it is unclear how lipid rafts in HSCs are affected by a HFD, systemic metabolic change may cause altered lipid metabolism of HSC’s plasma membrane, because it has been implied that dietary fatty acids, such as polyunsaturated fatty acids, affect membrane lipid raft organization [71]. Further investigation for molecular mechanisms of linkage between diet and HSC signaling is needed.

Another systemic effects of a HFD is abnormal intestinal permeability, setting the stage for plasma endotoxemia. Liu et al. reported that HFD-fed mice developed low-grade endotoxemia due to increased levels of serum LPS [63]. This report showed that feeding a HFD or treating with low-dose LPS induced functional changes in myeloid progenitors and CLPs, and that these changes depended on TLR4 signaling. However, the involvement of TLR4 signaling in HSC regulation under conditions of obesity is unclear. Since TLR4 activation by high-dose LPS is known to cause HSC dysfunction [72], it has been assumed that the endotoxemia resulting from HFD consumption may also impair HSC function. Detailed studies of the signaling molecules activated by low-dose bacterial pathogens in HFD-fed and obese animals are needed to clarify their effects on HSC regulation. These results indicate that the impact of HFD-derived factors on the regulation of HSCs and niche cells is in need of further exploration.

HFD consumption also changes the composition of the gut microbiota, often favoring the expansion of Gram-positive bacterial strains [73]. Recent studies have revealed that HFD-induced alterations to the composition of the gut microbiota affect hematopoietic homeostasis. For example, Luo et al. showed that the transfer of stool from HFD-fed mice to mice fed a ND caused the ND animals to exhibit HFD-associated hematopoietic and BM niche abnormalities. In the recipient mice, MPPs, myeloid progenitors, mesenchymal stem cells and adipocytes were all increased, whereas LT-HSCs, CLPs and osteoblasts were decreased. Cytokine expression levels were also altered in BM cells [74].

We and others have recently identified a critical effect of HFD on the self-renewal of HSCs in tumor-prone mice. We demonstrated that the altered gut microbiota composition caused by HFD affected the regulation of HSC self-renewal in Spred1-deficient mice, which develop myeloproliferative neoplasm-like disease and anemia. Spred1 is a negative regulator of RAS-MAPK signaling activated by SCF/c-Kit signaling [72]. Loss of Spred1 in ND mice enhanced HSC self-renewal capacity and success in cell competitions, suggesting that Spred1 negatively regulates HSC self-renewal. The consumption of HFD, but not LPS treatment or aging, induced ERK hyperactivation and aberrant self-renewal in Spred1-deficient HSCs, leading to the appearance of anemia and myeloproliferative neoplasm-like disease (Figure 3, right). Depletion of gut microbiota by antibiotic treatment mitigated these HFD-induced hematopoietic abnormalities, suggesting that the microbiota play a crucial role in regulating HSC homeostasis. Since downregulation of SPRED1 is observed in myeloid leukemia patients with a poor prognosis, [75], HFD-driven metabolic aberration or microbiota dysbiosis may significantly promote pathogenesis of leukemia patients.

### 3.4. The Effects of Metabolites Induced by Dietary Interventions on Stem Cell Fate Determination

Although our knowledge of how HSC regulation is affected by dietary interventions is currently limited, evidence of significant effects of various metabolites on other tissue stem cells has been accumulating. The next sub-section examines metabolites that control ISC self-renewal. The findings suggest possible roles of these metabolites in HSC homeostasis.

#### 3.4.1. Fatty Acid Composition

Previous reports showed that the treatment with either HFD or fatty acid constituents enhanced ISC self-renewal in a manner dependent on PPAR-fatty acid oxidation (FAO) in the absence of inflammatory signaling [76,77]. Conversely, intermittent fasting enhances self-renewal of ISCs by activation of PPARδ signaling through enhancing FAO [78]. These contradictory data suggest that fatty acids may be critical but complex regulators of HSC homeostasis.

#### 3.4.2. Ketone Bodies and Related Metabolites

During fasting, glucose levels decline and ketone biogenesis increases, releasing ketone bodies such as acetone and β-hydroxybutyrate (β-OHB) into the circulation. β-OHB enhances ISC self-renewal [79] by inhibiting histone deacetylases (HDACs) [80]. A ketogenic diet, which is a high-fat, low-carbohydrate diet, also induces the production of ketone bodies, which are used as alternate carbon sources. β-oxidation of fatty acids induced by a ketogenic diet promotes the activity of the tricarboxylic acid (TCA) cycle mediated by production of acetyl-CoA [81]. While there have been no reports of significant effects of a fasting or ketogenic diet on HSC behavior, it is plausible that such dietary interventions might modulate hematopoiesis and/or HSC aging.

#### 3.4.3. Microbiota Producing Metabolites

Alterations of the gut microbiota composition can induce systemic or local changes in gut microbiota-derived metabolites, which have recently been shown to affect organismal aging, tissue homeostasis and development of several diseases in humans as well as in mice [82,83,84]. For example, mice fed HFD show expansion of Gram-positive bacterial strains in the intestine. Increased levels of deoxycholic acid (DCA), which is a secondary bile acid produced by the microbiota, appear in the blood plasma of these animals. Bile acids, including secondary bile acids, are known to affect the regulation of HSC functions through their suppression of the ER stress response [85,86,87]. In addition, difference in intake of dietary fiber can change the spectrum of gut microbiota-derived metabolites in mice. Levels of short-chain fatty acids (SCFAs), including acetate, propionate and butyrate, are modulated by the feeding of dietary fiber, and these molecules can affect differentiation and the functions of immune cells [88,89]. Butyrate can also suppress the proliferation of murine ISCs in a FoxO3-dependent manner [90], implying an important role for this metabolite in regulating HSC biology. The same may be true for many other such metabolites in a broad range of stem cells.

## 4. Future Perspective

Over the past 15 years, there has been intensive study of the intracellular metabolic regulation in HSCs mediated by the anabolic/catabolic balance. The underlying rationale for these studies may be the hypothesis that the longevity or lifespan of an organism relies on the continued functionality of its stem cells. This body of work has now established that the activation of catabolic regulators is required for the maintenance of HSCs, as well as that of other tissue stem cells such as NSCs and MuSCs. Suppression of aberrant protein aggregation, mitochondrial activation, and oxidative stress has proven vital for the proper regulation of these tissue stem cells. Although mechanisms of control of HSC self-renewal by anabolic regulators have remained unclear, a deeper understanding of how stem cell behavior is regulated by the anabolic/catabolic balance will no doubt yield new strategies for maintaining healthy HSCs in vitro and in vivo.

The effects of catabolism on stem cell homeostasis in the context of cancer cell dormancy have not fully elucidated. Several catabolic regulators have been reported to play critical roles in the maintenance of stem cells in malignant tissues [91,92], but the functions of these regulators were found to be highly variable among cancers. Compared to tissue stem cells, cancer cells are much more complicated because they undergo numerous gene mutations and epigenetic alterations. It is thus difficult to find biological commonalities among such cells. However, cancer cell dormancy might be one area where a shared biological mechanism exists. It has recently been proposed that non-mutational drug resistance mechanisms underlie the survival of residual “drug tolerant persister” (DTP) cancer cells, and that these DTPs display transcriptional and functional similarities to cells in diapause, a reversible state of suspended embryonic development triggered by unfavorable environmental conditions [93,94]. Embryonic diapause is induced by inhibition of Myc [95] or mTOR [96], which are anabolic regulators. This finding suggests an important role for nutrient-sensing signaling in the survival of DTPs. That being said, this hypothesis appears paradoxical because the same signals have been thought to be important factors driving malignant progression. Further delving into how cancer behavior is influenced by nutrients may lead to the development of novel therapeutics useful for treating a variety of cancers.

Unlike the intensive analyses of intracellular metabolic regulators within HSCs, there has been little work performed to understand the molecular mechanisms by which changes to dietary nutrients affect HSC behavior. Although it is evident that the inflammatory machinery is at the center of this phenomenon, crucial players remain hidden. Specifically, studies of the effects of nutrients on cells in the BM niche supporting hematopoiesis and HSC homeostasis should be expanded, and the molecular mechanisms underlying the linkages between intracellular metabolism and systemic metabolic changes, and how they affect the microenvironments controlling stem cell behavior, should be explored. Many different cell types within the BM niche contribute to HSC homeostasis, including vascular cells, mesenchymal stem cells, mesenchymal lineage cells (osteoblasts and adipocytes), and neural cells. However, it remains unclear how these multiple cell types are organized by nutrient-related factors to preserve an environment suitable for healthy HSCs. To properly understand the spatiotemporal dynamics of HSC-niche interactions under various nutrient conditions, studies combining genomics, bioinformatics, metabolomics and imaging should be performed via interdisciplinary collaboration. Only by this multi-pronged approach will the mechanisms underlying the physiological roles of nutrients in the BM niche be clarified.

The knowledge gained from the above investigations may also spur the identification of compounds that can be used to influence HSC survival and behavior. It has been demonstrated that HSCs bearing genetic alterations to the *TET2*, *DNMT3A* or *ASXL1* genes exhibit abnormal differentiation with a bias toward myelopoiesis, promoting chronic inflammation. The myeloid-biased HSCs accelerate atherosclerosis and increase the risk of cardiovascular disease because of myeloid cells secretion of inflammatory cytokines or leakage of gut microbiota, both of which sustain systemic chronic inflammation. If compounds could be found to prevent the generation of aberrant HSCs despite their mutations, such products could have important medical applications in the future. Since these compounds will be natural molecules already present in the human body, it is likely that some may be useable without modification for the prevention of hematopoietic abnormalities, such as aging-related myelodysplasia. In addition, the early detection of upregulation of a metabolite known to be harmful to HSCs could aid in the diagnosis of hematopoietic diseases. While genetic alterations are critical indicators, additional information with biomarkers that reflect inflammatory status would contribute to accurate prediction of prognosis and successful medical intervention.

In conclusion, continued research in the field of the effects of intra- and extra-cellular metabolism on HSCs is both necessary and potentially highly rewarding. These efforts will no doubt make numerous major contributions to society in the form of new tools for medical research and novel therapeutic agents for improving human health care.
